# Effects of Surface Treatments of Glass Fiber-Reinforced Post on Bond Strength to Root Dentine: A Systematic Review

**DOI:** 10.3390/ma13081967

**Published:** 2020-04-23

**Authors:** Lora Mishra, Abdul Samad Khan, Marilia Mattar de Amoedo Campos Velo, Saurav Panda, Angelo Zavattini, Fabio Antonio Piola Rizzante, Heber Isac Arbildo Vega, Salvatore Sauro, Monika Lukomska-Szymanska

**Affiliations:** 1 Department of Conservative Dentistry and Endodontics, Institute of Dental Sciences, Siksha ‘O’ Anusandhan Univeristy, Bhubaneswar 751003, India; loramishra@soa.ac.in; 2 Department of Restorative Dental Sciences, College of Dentistry, Imam Abdulrahman Bin Faisal University, Dammam 31441, Saudi Arabia; akhan@iau.edu.sa; 3 Department of Operative Dentistry, Endodontics and Dental Materials, Bauru School of Dentistry, University of São Paulo, São Paulo 17012-901, Brazil; mariliavelo@usp.br; 4 Department of Periodontics and Oral Implantology, Siksha ‘O’ Anusandhan Univeristy, Bhubaneswar 751003, India; sauravpanda@soa.ac.in; 5 Department of Biomedical, Surgical and Dental Sciences, Universita Degli Studi di Milano, 20122 Milano, Italy; 6 Department of Endodontics, King’s College London Dental Institute, Guy’s Hospital, London SE1 9RT, UK; angelo.zavattini@kcl.ac.uk; 7 Department of Comprehensive Care, School of Dental Medicine, Case Western Reserve University, Cleveland, OH 44106, USA; fap17@case.edu; 8 Department of General Dentistry, Dentistry School, Universidad San Martín de Porres, Chiclayo 14012, Peru; harbildov@usmp.pe; 9 Department of General Dentistry, Dentistry School, Universidad Particular de Chiclayo, Chiclayo 14012, Peru; 10 Dental Biomaterials, Preventive and Minimally Invasive Dentistry Departamento de Odontología, Facultad de Ciencias de la Salud, Universidad CEU-Cardenal Herrera C/Del Pozo ss/n, Alfara del Patriarca, 46115 Valencia, Spain; salvatore.sauro@uchceu.es; 11 Department of Therapeutic Dentistry, Sechenov University of Moscow, Mozhaisky Val 11, 119435 Moscow, Russia; 12 Department of General Dentistry, Medical University of Lodz, 92-213 Lodz, Poland

**Keywords:** glass fiber, composite post, endodontic therapy, etching, post and core technique, silane

## Abstract

The objective of this systematic review was to determine the influence of surface treatment of glass fiber posts on bond strength to dentine. Laboratory studies were searched in MEDLINE, PubMed, Embase, PubMed Central, Scopus, and Web of Science search engine. All authors interdependently screened all identified articles for eligibility. The included studies were assessed for bias. Because of the considerable heterogeneity of the studies, a meta-analysis was not possible. Twelve articles were found eligible and included in the review. An assessment of the risk of bias in the included studies provided a result that classified the studies as low, medium, and high risk of bias. The available evidence indicated that the coronal region of the root canal bonded better to the glass fiber post than apical regions. Phosphoric acid, hydrogen peroxide, and silane application enhance post’s retentiveness. In light of the current evidence, surface treatment strategies increase the bond strength of glass fiber post to dentine. However, recommendations for standardized testing methods and reporting of future clinical studies are required to maintain clinically relevant information and to understand the effects of various surface treatment of glass fiber post and their bond strength with dentine walls of the root canal.

## 1. Introduction

It has been advocated that teeth become more fragile after endodontic treatments, and this increases the failure rate of post-endodontics restorations [1] due to a loss of tooth structure, moisture, and flexibility of the dentine [2]. Therefore, extensive tooth loss after cavity preparation represents a real challenge in the restoration of endodontically treated teeth. Thus, in such a specific clinical scenario, the use of intra-canal posts may be indicated to increase the retention of the core and/or of the coronal restoration [3].

Recently, several different posts and types of core materials are in use, whereby fiber-reinforced posts (FRC) have been used with acceptable results both in clinical practice and in research [4,5]. Due to “dentine-like” elastic modulus and exceptional esthetic properties, FRC has shown superior performance compared to metal posts [2,6]. Moreover, the adhesive bond between FRC and resin cement provides a short term strengthening effect, theoretically creating an endodontic “Monoblock” [7,8]. Nevertheless, bonding posts to root canal dentine can be compromised [4,9]. The limitations are usually related to polymerization effectiveness [9], difficult in creating a water-wet substrate [10,11,12,13], reduced number of dentinal tubules [11,14,15], deposition of cementum [10,12] and secondary dentin [13]. As bonding to root dentine is still unpredictable, it may affect the longevity and the clinical performance of the restorations [3,16,17].

The stability of the resin-root dentine interface is also affected by the presence of the endogenous enzymes activated during etching procedures. These are in part responsible for the hybrid layer’s degradation and reduction of the longevity of post-endodontists restorations performed with the use of root canal posts [18,19]. Various efforts to enhance the longevity of the interface between root canal dentine and FRC posts have been advocated. Such strategies include adhesive treatment, tribomechanical treatment, sandblasting, as well as a combination of these methods (Co-Jet) [20,21,22].

Despite the existence of some positive results in vitro [23], the treatment of the surfaces of posts is still considered a technique-sensitive procedure, and there is a lack of long-term clinical studies.

To the best of our knowledge, there is no standard protocol for post-space treatment and FRC surface treatment that can assure long-term performance in both clinical and in vitro scenarios [3]. The wide variety of materials available in the market, associated with the limitations of bonding to root canal dentine, can interfere with the adhesive strategy. Therefore, it is essential to investigate the long-term stability of the interface between fiber posts and adhesive materials to guide clinicians to select the most appropriate materials and treatment protocols in dental practice. The present systematic review aimed at appraising the existing literature on the effect of post-treatment on the bonding of fiber-reinforced composite posts to root dentin.

## 2. Materials and Methods

**Figure 1:**
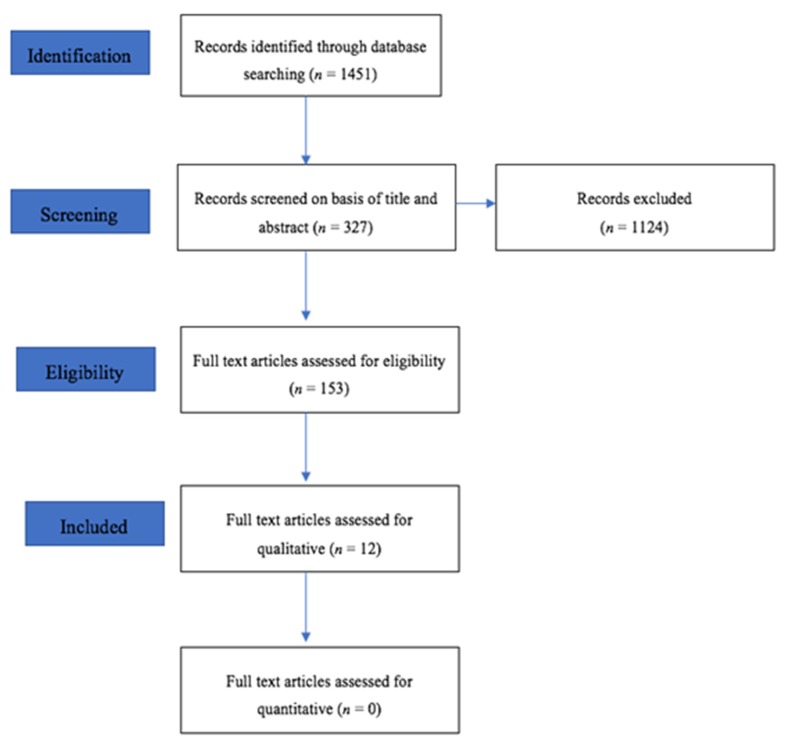
PRISMA flow diagram of the literature search and selection process. PRISMA—Preferred Reporting Items for Systematic Reviews and Meta-Analyses.

This review was carried out following the Preferred Reporting Items for Systematic Reviews and Meta-Analyses (PRISMA) statement guidelines as reported previously [24]. The schematic pattern of the protocol is shown in Figure 1.

### 2.1. Search Strategy

The initial review carried out with a MEDLINE/PubMed, EMBASE, CENTRAL, Scopus, and Web of Science search with laboratory and clinical trial findings. The structured protocol was adopted for the search and the following keywords were: (“silane” or “treatments” or “tissue conditioning” or “dental etching” or “dental cements” or “bond strength”) and (“fiber post” or “glass fiber post” or “post and core technique” or “fiber-reinforced post” or “endodontic post”) and (“root dentin” or “dentin” or “tooth root” or “root canal”).

### 2.2. Study Selection

The search of the literature was performed without any restriction on a date and was done up to June 2019. Full texts of papers were obtained from the journals. The inclusion and exclusion criteria for articles are presented in Table 1.

### 2.3. Study Quality Assessment

The title and abstracts of all articles identified by the electronic search were read and assessed by two authors (S.P. and F.A.). Disagreements between the reviewers were resolved by consensus with all the authors. The methodological quality of all selected full-text articles was assessed using the guidelines reported previously [1,24]. The description of the parameters for quality assessment was: randomization of teeth, use of teeth free of caries or restoration, use of materials according to the manufacturer’s instructions, use of tooth roots with similar dimensions, endodontic treatment performed by the same operator, description of sample-size calculation, and blinding of the operator of the testing machine. If the parameter was mentioned in the article, a “positive” (+) sign was assigned to that specific parameter; if the information was not provided, the article received a “negative” (−). The searched articles that reported one to three parameters were classified as having a high risk of bias, four or five items as medium risk of bias, and six or seven items as low risk of bias.

After the application of the search strategy, two examiners (L.M. and M.V.) reviewed and performed the selection by consensus to complement the database searches. References in papers were checked and cross-matched with those from the original search. Where additional references were found to meet the inclusion criteria, then these were included in the review. After identifying the eligible studies in the above databases, these studies were imported into Endnote X7 software (Thompson Reuters, Philadelphia, PA, USA) to remove duplicates.

## 3. Results

After the exclusion of duplicated articles, the electronic search identified 1451 articles from EMBASE, PubMed/ Medline, Scopus, and Web of Sciences. However, 327 articles were initially selected based on titles and abstracts, whereby 153 articles appeared as full text. The total number of papers, which met the inclusion criteria for the review, was 12. The flow diagram (Figure 1) depicts the details of the search strategy and Table 2 lists out surface treatment and bond strength analysis of FRC posts.

### 3.1. Pre-Treatment of FRC Post Surface with Silane

Among these studies, six articles [22,25,26,27,28,29] evaluated the effect of surface treatment with silane on the bond strength to dentine. Whereby, four studies [22,25,26,27] evaluated the push-out bond strength, and the other two assessed the retentive bond strength [28,29]. The push-out bond strength was evaluated in coronal, middle, and apical regions [25,26,27], whereas, only one study [22] reported the mean values of all three areas together. Only two studies [25,27], compared silane with the control group. All studies concluded that silane alone could not enhance the bond strength of FRC post and resin cement to dentine. Two studies [28,29] evaluated the pull-out bond strength between silane and control groups and reported that the silane treatment of fiber posts did not prevent dislocation.

### 3.2. Pre-Treatment of FRC Post Surface with Air-Borne Particle Abrasion

The search data showed that alumina particles were used to treat FRC post surface [25,27,28,30,31], whereby the used particle size was mainly 50 μm. Only one study [28] assessed pull-out bond strength by using 30 µm particle size. In a few studies [26,27,28], silane was applied after air abrasion pre-treatment.

Air-borne particle treatment showed significantly lower push-out bond strength compared to the control group in the coronal and middle levels of the root [25,26,27]. Nevertheless, in another study [30], the mean results (from the three root regions), showed a higher bond strength than the control group. Two studies performed retentive and pull-out bond strength tests [31,32] and found that air-borne particle treatment group performed better than the control group. In one of those two studies [31], posts received additional aging treatment followed by the bonding agent application.

### 3.3. Pre-Treatment of FRC Post Surface with Hydrofluoric Acid

In one study [25], push-out bond strength was evaluated in the coronal, middle, and the apical section of the roots after treating the fiber post with hydrofluoric acid, however, found low bond strength values when compared to control group. Other studies [26,33], reported the push-out bond strength values of hydrofluoric acid-treated glass fibers post followed by silane application and showed the non-significant difference in values with the control group. Nevertheless, posts treated with hydrofluoric acid and silane followed by heat treatment showed significantly increased bond strength in comparison to the control group [33]. Another study [28], reported low pull-out bond strength values of hydrofluoric acid-treated glass fiber posts compared to control, silane treated, and sandblasting with silane groups.

### 3.4. Pre-Treatment of FRC Post Surface with Laser

It was observed in two selected studies [26,30] that Nd:YAG laser with different pulse durations in combination with silane increased the surface roughness, however, no significant difference was found in push-out bond strength between the control and surface treated groups [26]. In particular, the femtosecond laser resulted in lower bond strength when compared to control and air-borne particle abrasion groups [30]. Another study [32] evaluated pull-out bond strength of pre-treated posts using a 1.5-, 3-, and 4.5-W Er:YAG laser and it was found that the 4.5-W laser had higher values compared to air-abrasion and the control group (no treatment).

### 3.5. Pre-Treatment of FRC Post Surface with Etching Agents

In one of the selected studies [25], it is reported that etching the fiber post surface using CH_2_Cl_2_ could improve the bond strength values compared to hydrofluoric acid, air-abrasion, silane treated, and the control group. The use of 37% phosphoric acid for the different periods increased the pull-out and push-out bond strength of the posts bonded to root dentine compared to the control group, especially when phosphorous acid was used for 15 s [21,31]. In one of the studies [21], push-out bond strength was evaluated in the coronal, middle, and apical sections of roots after treating the fiber post with 37% phosphoric acid, however, indicating low bond strength values when compared to control group.

### 3.6. Pre-Treatment of FRC Post Surface with Hydrogen Peroxide (H_2_O_2_)

The highest bond strength values in both push-out tests were observed after 60 s application of H_2_O_2_ on glass fiber post in comparison to 15 s, 30 s, and the control group [21]. Pre-treatment of the post with 20% hydrogen peroxide for 20 min before silanization resulted in higher push-out bond strength at all of the three root regions compared to air-abrasion with silane, silane alone, and the control groups [27]. Likewise, treatment of glass fiber posts with 10% hydrogen peroxide for 20 min followed by silane and additional heat treatment showed greater push-out bond strength in all regions of the root compared to the specimens that received no heat treatment [33].

### 3.7. Pre-Treatment of FRC Post Surface with Alcohol

Two searched studies [29,34] evaluated the pre-treatment of post surfaces with alcohol. One study [29] showed that the post’s surface pre-treatment with 21.75% ethanol had lower performance compared to the post-treated with silane, however, performed better in comparison to the non-treated control group. The other study [34] assessed the effects of post’s surfaces pre-treatment with 96% alcohol with and without conditioning primer application. It was concluded that the alcohol group and alcohol with primer group exhibited the least retentive forces compared to the air-abrasion group and the combination of all three pre-treated groups.

### 3.8. Pre-Treatment of FRC and the Artificial Aging Process

Only one study [31], compared the effects of aging on the surface of treated specimens. Groups that underwent phosphorous acid followed by aging showed increased bond strength values when compared to the non-aged groups.

### 3.9. Risk of Bias Analysis

**Figure 2:**
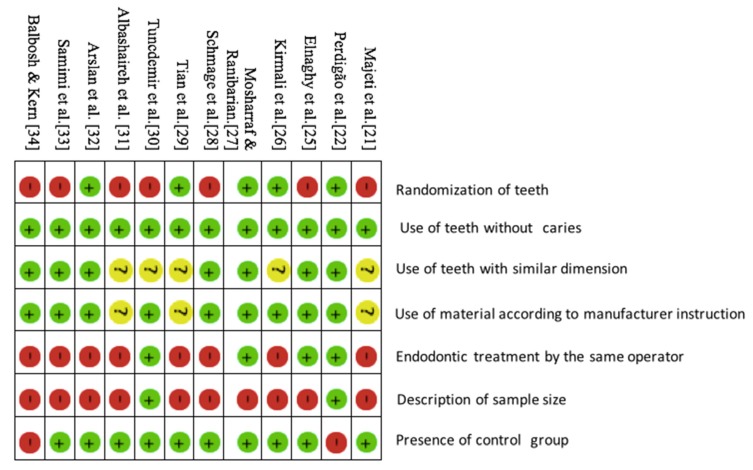
Risk of bias.

Five studies [21,28,29,31,34] were classified as high risk, five studies [25,26,30,32,33] were classified as medium risk, and only two studies [22,27] were classified as low risk. The outcome of the risk of bias analysis is presented in Figure 2. It was found that six studies [21,25,28,30,33,34] did not mention randomization of teeth, eight studies [21,26,28,29,31,32,33,34] did not mention either experimental procedure was performed by single operator or multiple operators. It was searched that eleven studies [21,22,25,26,27,28,29,31,32,33,34] did not mention a description of sample size and two studies [26,34] did not use control group; five studies [21,28,29,30,31] did not report about length/dimension of the root; and six studies [21,26,29,30,31,34] did not report that either sample was used according to manufacturer’s instructions or not.

## 4. Discussion

Different strategies have been adopted to enhance the bond strength between the FRC, resin cement, and dentine, as it enables FRC to resist vertical dislodging forces. A high ratio of crosslinking within the polymer matrix makes it unable to reactivate, and negatively affects the bonding of FRC post assembly to dentine [32].

One of the strategies to improve the interfacial bond strength consists of the treatment of the FRC surface before bonding. The plethora of pretreatments of the FRC surface was noted and qualitatively analyzed in this review. Nevertheless, no consensus for surface treatment was seen that can be universally adopted. The bond strength was mainly assessed by push-out and pull-out test methods.

Studies that did pull-out were justified as a more significant methodology to analyze bond strength between fiber posts and root canal dentin [32]. In push-out studies, posts in three regions of roots (coronal, medial, and apical) were tested mainly and least bond strength was observed in the apical region [21,25,26,27,33]. Whereas, Finite Element Analysis (FEA) data showed homogeneous stress distribution around the post circumference in these regions. The factors related to low bond strength in the apical region could be inefficient removal of smear layer due to lack of accessibility of instruments and irrigating solution, lack of flow of material in constricted space, which results in void and bubble formation, and incomplete curing of material as the light source does not reach that region effectively [35].

It is difficult to relate in vitro condition with actual clinical situation, whereby teeth are subjected to compressive occlusal load along with shear and bending forces. The distribution of these loads and forces are different on anterior and posterior teeth [22,36,37]. The authors could not find any in vitro study in this review search where all these forces and loads were applied on endodontic posts and restoration to simulate the more clinically relevant situation. In this review, only in vitro studies conducted on human teeth were included because inconsistent data exist in the literature regarding bovine teeth. It is inconclusive that bovine teeth should be considered as an appropriate substitute for human teeth or not [38].

Many studies claimed to apply a silane agent on the post to enhance the bonding of post to resin cement and dentine [11,39]. Its application is effortless and easy, making it a widely accepted pre-treatment procedure among researchers. However, the effect on bond strength to dentine is unconvincing [22,25,26,27,28,33,40], which might be due to the manual application of silane on the post surface and formed a non-homogenous layer, subsequently led to a weak chemical bond between silane and post [25]. Another reason could be the composition of the resin matrix of FRC posts. It is reported that the methacrylate matrix bonds better to silane solution than epoxy resin containing FRC as depicted in Table 3 [21,22,25,29,40]. The interface between resin cement and post was also considered as a contributing factor for mechanical strength, where limited mechanical interlocking was mentioned between resin cement and untreated post surface [30,31]. Surface treatments such as air-borne abrasion, etching, laser treatment, hydrogen peroxide, etc. altered the surface topography and increased the retention of the FRC post.

Air abrasion is a technique capable of altering the surface topography by partially removing the matrix of the post and creating retention spaces without jeopardizing their mechanical properties [25,26,30,31,41]. However, the reported data showed that the air-abrasion treatment showed significantly lower push-out bond strength compared to the control group in the coronal and middle levels of the root [25]. Nevertheless, in another study [27], a higher push-out bond strength was observed than in the control group. Whereas, a significant reduction of bond strength was found at the apical part when compared with the middle and coronal parts [25,27]. The pull-out bond strength tests [28,32] concluded that air-borne abrasion performed better than the control group. The heterogeneity in the reported results could be due to the differences in particle size, pressure, time, and distance. Distance and time of exposure are critical for the beneficial effect of air-borne abrasion on the FRC post [30,31,32,34]. Longer abrasion time damages the fibers and reduces the diameter and compromises the fit of the post to the dentine [25,26,27,28].

Usually, single surface treatments are not capable of creating strong bond strength between post and resin. Additional etching techniques including mechanical and chemical were rendered to FRC post [25,27]. Micromechanical procedures roughen the post surface and provide access to silane solution and bonding primer agents to have more intimate contact and interaction with fibers [35,41]. Surface activation procedures for the post would be a benefit, and maybe this is one of the reasons that sandblasting followed by silane and primer application performed better than non-etched groups [27,28,31,34]. Other surface treatments, such as 9% hydrofluoric acid resulted in the dissolution of resin matrix at greater depth and also extensively damaged glass fibers within the post, therefore, reduced bond strength values were observed when compared to untreated and other experimental groups [25,26].

Laser parameters in terms of intensity, frequency, wavelength, and ablation rate are the deciding factors to obtain optimal bond strength and roughness values. This could be one of the reasons for the better performance of post irradiated with 4.5-W Er:YAG laser compared to other experimental groups [32]. However, the high power setting of laser etching can damage the fibers and produce inconsistent surface changes [17,23,42].

Many protocols directed towards surface treatments selectively remove the superficial epoxy resin layer but maintain the integrity of the fibers. Most commonly available etchants i.e., 37% phosphoric acid and hydrogen peroxide can facilitate the micromechanical retention of resin cement to the post, achieving the desired results. Two studies have recommended the application of phosphoric acid and hydrogen peroxide for 15 s and 60 s, respectively [21,31]. Aggressive/longer etching can affect the fiber’s integrity [21]. Prolonged use of H_2_O_2_ can jeopardize resin polymerization due to the formation of free radicle and oxygen-rich layer, resulting in low bond strength [14,43]. A newer agent like methylene chloride (CH_2_Cl_2_) showed enhanced interfacial bond strength between the post and the luting agents. It removed the superficial layer of the resin matrix and exposed the fibers for better retention results [25].

There is a considerable amount of disparity in the retention and bond strength values of surface-treated FRC posts. This heterogeneity of observed values could be a result of not reproducibility of a clinical scenario [28]. The thermo-cycling or aging process is one of the ways to evaluate the bond strength of post in a simulated clinical environment [25]. The average number of thermal cycles that would normally occur in the oral cavity ranges from 4000 to over 10,000 per year [44]. The lack of uniformity was observed in the number of cycles and the period to which specimens were exposed [27,28,31,33,34]. Nevertheless, in a clinical scenario, FRC posts placed in the root, surrounded by dentine and alveolar bone; therefore, they remain unaffected by the temperature changes. It is reported that the thermo-cycling in water weakened the FRC posts and reduced their flexural modulus [45]. This also could be one of the reasons for reduced bond strength when the post was directly exposed or stored in water [28,31,33,34]. Other reasons for inconsistency in results among in vitro studies could be the storage time (24 h and 1 month) of FRC posts, resin, and dentine assembly in distilled water, which is not the true representative of the clinical scenario [22,25,26,27,28,29,30,31,32,33].

The overall quality of selected in vitro studies for qualitative analysis was poor. Most studies did not justify the sample size selection and did not mention that the endodontic treatment was carried out by the same operator. Another factor that contributed to low evidence of available data is that teeth were not randomly distributed into the groups. The high heterogeneity of the included studies prevented a quantitative analysis of data. Only two studies [22,27] out of 12 had a low risk of bias according to the used quality assessment criteria. Therefore, any general conclusions need to be drawn cautiously. Another limitation was the language restriction placed in the search strategy. There might be good quality in vitro studies in languages other than English which could have influenced the outcome of this review. In addition, the effect of luting cement on bond strength which forms an interface between post and dentine has not been included in this review. The limitation of the present review is that the influence of the shape and design of the post was not taken into account.

### Future Consideration

There is a need for good quality in vitro studies which should at least try to simulate clinical conditions. FRC post-pre-treatment compared to other aesthetic posts like zirconia, can be evaluated in future studies. In addition, the effect of dentine conditioning and pre-treatment procedures influencing the bonding assembly of FRC posts can be evaluated in further reviews and research. More studies are required to analyze the efficacy of methylene chloride and ER:YAG laser pre-treatments of FRC post.

## 5. Conclusions

Based on this systematic review, the following conclusions were drawn:

1. The coronal region of the root canal exhibits higher bond strength to the FRC post than apical regions.
2. Phosphoric acid and hydrogen peroxide are the rapid chairside technique which can be used to increase post retention.
3. Micromechanical treatment followed by silane application can result in optimum bond strength of FRC post to resin cement and dentin.
4. Most of the studies reported that silane application to the epoxy resin-based FRC post did not enhance the bond to dentin.
5. It is not recommended to use hydrofluoric acid to treat the FRC post, as it extensively damages the surface topography of the post.

## Figures and Tables

**Table 1:** The inclusion and exclusion criteria for articles.

Inclusion Criteria	Exclusion Criteria
- In vitro studies carried out on radicular dentin of human extracted teeth. - Surface treatment of radicular dentin was performed after removing the obturating material and prior to restoring with glass fiber posts. - Studies based on glass fiber endodontic posts. - The push-out and pull-out test was performed to evaluate the bond strength.	- In vivo studies and in vitro studies performed on animal models. - Studies testing endodontic posts other than glass fibers, i.e., carbon and metal posts. - Literature not published in peer-reviewed journals. - The grey literature, i.e., the information not reported in the scientific journals. - All papers in a foreign language (not in the English language), where the full text was not available.

**Table 2:** Surface treatment strategies done on the fiber-reinforced (FRC) post.

Serial Number	Pretreatment Technique	BS Analysis	References
1	S	PBS	[22,25,26,27]
2		RBS	[28,29]
3	AP	PBS	[25,30]
4		RBS	[31]
5		Pull out	[32]
6	AP + S	PBS	[26,27]
7		RBS	[28]
8	HF + S	PBS	[26,33]
9		Pull out	[28]
10	AP + Alc	RBS	[34]
11	AP + BA (Ag/no Ag)	RBS	[31]
12	HF	PBS	[25]
13	Laser	PBS	[26,30]
14		Pull out	[32]
15	H_2_O_2_	Push out	[21]
16	H_2_O_2_ + S	PBS	[27,33]
17	AE	PBS	[21,31]
18	AE + BA	RBS	[31]
19	Alc, Alc + primer	RBS	[34]
20	Ethanol + S	RBS	[29]
21	CH_2_Cl_2_	PBS	[25]

BS—bond strength; PBS—push out bond strength; RBS—retentive bond strength; S—silane; AP—airborne abrasion; Alc—alcohol; BA airborne abrasion application followed by bonding agent application; HF—hydrofluoric acid; H_2_O_2—_hydrogen peroxide; AE—acid etching; CH_2_Cl_2—_methylene chloride.

**Table 3:** Composition of different FRC post used in the selected invitro studies.

Serial Number	Type of Post	Matrix	Filler	References
1	Rebilda post	Dimethacrylate	Glass	[25,26,32]
2	FRC post			[22,33]
3	Hetco fiber post	Epoxy		[27]
4	Radix			[31]
5	Dentin post ER			[28,34]
6	Para post			[22]
7	Glassix posts Nordin			[21]
8	DT light post		Quartz	[22,29,30]
